# Serum osteoglycin is stable during various glycemic challenges in healthy men

**DOI:** 10.1007/s12020-024-03789-1

**Published:** 2024-03-28

**Authors:** Jakob Starup-Linde, Sidse Westberg-Rasmussen, Rikke Viggers, Zheer Kejlberg Al-Mashhadi, Aase Handberg, Peter Vestergaard, Søren Gregersen

**Affiliations:** 1 Department of Endocrinology and Internal Medicine, Aarhus University Hospital, Central Region Denmark, Aarhus N, 8200 Denmark; 2 Steno Diabetes Center Aarhus, Aarhus University Hospital, Aarhus, Denmark; 3 Steno Diabetes Center North Denmark, Aalborg University Hospital, Aalborg, Denmark; 4 Department of Clinical Medicine, Aalborg University, Aalborg, Denmark; 5 Department of Clinical Biochemistry, Aalborg University Hospital, Aalborg, Denmark; 6 Department of Endocrinology, Aalborg University Hospital, Aalborg, Denmark

**Keywords:** Osteoglycin, Glucose, Oral glucose tolerance test, Insulin, Isoglycemic intravenous glucose infusion

## Abstract

**Purpose:**

Osteoglycin is hypothesized to be metabolically active and may enhance insulin action. We hypothesized that osteoglycin levels increase during hyperglycemia as a physiological response to enhance the effects of insulin.

**Methods:**

Eight healthy males were included in a cross-over study consisting of three study days following an 8 h fast. First, we performed an oral glucose tolerance test (OGTT); second, an isoglycemic intravenous glucose infusion (IIGI); and third, a control period consisting of a three hour fast. We analyzed blood samples for circulating osteoglycin levels during the study days. Repeated measures ANOVA was performed to compare levels of s-osteoglycin between OGTT, IIGI, and the fasting control.

**Results:**

There were no differences in baseline osteoglycin levels among study days (*p* > 0.05). We observed no significant changes neither in absolute s-osteoglycin levels by time (*p* = 0.14) nor over time by study day (*p* = 0.99). Likewise, we observed no significant changes in percentage s-osteoglycin levels neither by time (*p* = 0.11) nor over time by study day (*p* = 0.89).

**Conclusion:**

We found that s-osteoglycin levels were stable for three hours during OGTT, IIGI, and fasting in healthy males. Based on the present study, circulating s-osteoglycin levels may be measured independently of fasting or non-fasting conditions. Furthermore, circulating physiological levels of glucose and insulin did not affect s-osteoglycin levels.

## Introduction

Osteoglycin is a proteoglycan that derives from bone, cartilage, and muscle and is hypothesized to have endocrine effects on bone and pancreas [1]. In a study in mice treated with osteoglycin, a dose-dependent lowering of blood glucose was found [2]. In the same study, treatment with osteoglycin induced a significantly larger decline in glucose levels in response to insulin during an insulin tolerance test. Mice with a knockout for the osteoglycin gene presented impaired glucose tolerance and high amounts of white adipose tissue compared to wild-type mice [2]. These findings suggest that osteoglycin in mice enhances the effect of insulin.

In humans, osteoglycin levels increase following severe weight loss induced by sleeve gastrectomy, gastric bypass surgery, or dietary intervention and are associated with the glycemic response to weight loss, which is suggestive of metabolic effects [2]. Individuals with type 2 diabetes have higher levels of osteoglycin compared to those without diabetes [3]. However, in persons with type 1 diabetes or type 2 diabetes, s-osteoglycin levels were not associated with p-glucose independent of fasting state or glycated hemoglobin A1c (HbA1c) levels [4, 5]. A post hoc analysis of a cross-over trial in which participants exercised or rested followed by a standardized meal showed that exercise had limited effects on s-osteoglycin levels [6]. In addition, it was found that a meal following exercise did not alter s-osteoglycin levels, whereas a meal following rest resulted in a decline in s-osteglycin at time points 90 and 120 min after the meal. However, this study did not provide a fasting control group [6]. Another study reported that s-osteoglycin levels were positively associated with p-glucose in individuals without diabetes [7]. Thus, conflicting results exist regarding the role of circulating osteoglycin in humans.

In the present study, we examined whether s-osteoglycin levels change in response to different glycemic challenges by oral glucose tolerance test (OGTT), an isoglycemic intravenous glucose infusion (IIGI), and a fasting control state that elicit different responses in insulin and incretin hormones. We have previously reported higher levels of insulin and incretin hormones during an OGTT compared to the IIGI, and the IIGI led to higher levels of insulin but not of incretin hormones compared to the fasting control [8]. We hypothesized that s-osteoglycin levels increase during hyperglycemia to enhance the effect of insulin.

## Methods

The present study is a posthoc analysis of a crossover study with three study days. The original study has been described elsewhere [8]. The study is approved by the Danish Data Protection Agency (2007- 58-0010) and the Ethics Committee of the Central Denmark Region (1-16-02-377-13).

In brief, twelve healthy Caucasian males, aged 20–50 years, were recruited by postings at Aarhus University and online at www.forsøgsperson.dk. All gave informed consent to the study. Individuals with possible bone-disorders, including osteoporosis, diabetes mellitus, and thyroid disorder, were excluded. The study was conducted as a crossover study with two interventions on two separate days at least one week apart. After completing both interventions, participants were asked to participate in a three-hour fast. Eight of the twelve participants accepted enrollment in the three-hour fast, and were included in the present study. Thus the study days consisted of; day one: OGTT, day two: IIGI, and day three: Three hours fast. The day before each study day, participants were asked to refrain from performing physical exercise, smoking, and taking vitamin supplements. A standard meal was delivered by the clinic to be ingested between 5 and 11 PM on the evening prior, and participants were asked to fast (water allowed) from 11 PM until the following morning. They were asked to arrive by car or bus to the clinic.

On study day one an OGTT was performed. The OGTT consisted of an oral solution of 82.5 g of glucose monohydrate (equivalent to 75 g of D-glucose), 225 ml water and 225 mg benzoic acid. Blood glucose levels were monitored every 5 min during the first two hours by Accu-Chek Inform II apparatus (Roche Diagnostics, Basel, Switzerland). On study day two an IIGI was performed. During the IIGI, 20% D-glucose was infused in a cubital vein to mimic the plasma glucose curve obtained during the OGTT. Infusion rates for the glucose solution were adjusted according to the measured plasma glucose level. On study day three a three-hour fast was monitored. During the fasting control, only collection of blood samples from participants occurred.

Blood samples were collected at times 0, 60, 120, and 180 min during each study day.

Blood samples were frozen and stored at minus 80 °C. Osteoglycin was analyzed on serum by an ELISA-assay from Cloud-Clone Corp (Wuhan, China) essentially as described by the provider. In brief, samples were analyzed in duplicates in a 1:10 dilution in PBS (phosphate-buffered saline). All samples from a participant were always analyzed on the same day. Assay control from Cloud-Clone Corp (Wuhan, China) and a serum pool were both analyzed in duplicates at each run. Mean percent coefficient of vartiation (CV%) of the duplicates was 4.9%. Mean CV% between runs was 17.4% for assay control (4 runs) and 20.7% on serum pool (4 runs), whereas mean CV% on samples re-analyzed in duplicates was 13% (2 runs).

Plasma insulin was measured by ELISA using a DAKO insulin kit (Code: K6219; Dako, Glostrup, Denmark). Analysis of gastric inhibitory peptide (GIP), glucagon-like peptide-1 (GLP-1), and glucagon-like peptide-2 (GLP-2), have previously been described [8].

Serum procollagen type I N propeptide (s-P1NP), and serum C-terminal telopeptide of type I collagen (s-CTX) are used as reference bone turnover markers in clinical studies [9]. s-CTX and s-P1NP were measured by immunometric sandwich assays using the COBAS 6000 E (Roche Diagnostics, Basel, Switzerland). The CV% for the analyses are 5 and 3.7%, respectively.

Statistical analysis was carried out using the STATA 17 package (StataCorp, College Station, Texas, USA). Repeated measures ANOVA was performed to compare levels of s-osteoglycin between OGTT, IIGI and the 3-hour fasting control.

Normality of data was checked via qq-plots. We performed three conservative F-tests to ensure validity of results.

Homeostatic model assessment of insulin resistance (HOMA-IR) and Homeostatic model assessment of beta cell function (HOMA-β) were calculated as (glucose x insulin)/22.5 and insulin x 20/(glucose-3.5), respectively. Using a simple linear regression model, the association between the baseline s-osteoglycin levels and baseline levels or changes from time point 0 to time point 60 min of p-glucose, p-insulin, HOMA-IR, HOMA-β, p-GIP, p-GLP-1, p-GLP-2, s-CTX, and s-P1NP were examined.

No power calculation was performed on osteoglycin as this study is a posthoc analysis of the primary study in which CTX was the primary endpoint.

## Results

Eight healthy males with an age ranging between 25 and 48 years and all non-smokers were included in the present study. The characteristics of the included subjects are presented in Table 1.

**Table 1:** Baseline characteristics at inclusion to the study (mean, range)

	*N* = 8
Age (years)	30 (25; 48)
BMI (kg/m^2^)	24 (22; 29)
Systolic blood pressure (mmHg)	134 (121; 155)
Diastolic blood pressure (mmHg)	78 (70; 93)
HbA1c (%)	5.3 (4.8; 5.7)
HbA1c (mmol/mol)	34 (29; 39)
25 hydroxy vitamin D (nmol/l)	57 (42; 66)
Ionized calcium (mmol/l)	1.22 (1.17; 1.28)

*OGTT* oral glucose tolerance test, *IIGI* intravenous isoglycemic glucose infusion

**Fig. 1:**
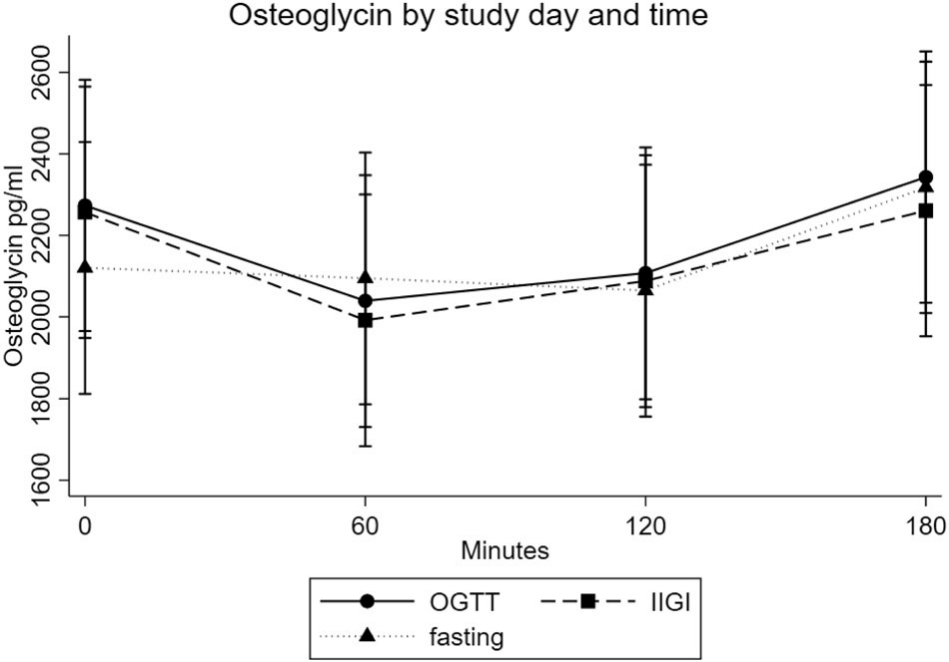
s-osteoglycin levels by study day and time. OGTT Oral glucose tolerance test, IIGI intravenous isoglycemic glucose infusion. The error bars present 95% CI

**Fig. 2:**
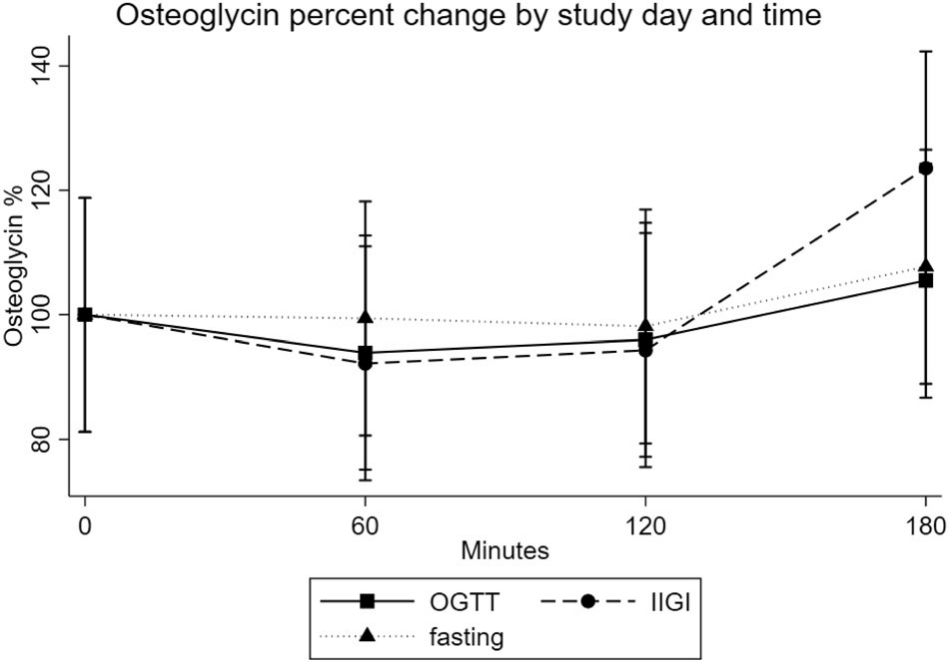
Percentage s-osteoglycin levels by intervention and time. OGTT Oral glucose tolerance test, IIGI intravenous isoglycemic glucose infusion

The baseline osteoglycin levels were not significantly different among study days (*p* > 0.05) (see Table 2). Figure 1 illustrates absolute changes in osteoglycin levels during the study days. Figure 2 illustrates percentage change in s-osteoglycin levels from baseline during the study days. We observed no significant changes in absolute s-osteoglycin levels neither by time (*p* = 0.14) nor over time by study day (*p* = 0.99). When visually assessing the changes in s-osteoglycin on individual level, we still observed no changes by time or study day (results not shown).

**Table 2:** Baseline osteglycin, glucose, and insulin at each study day (mean, 95%CI)

	IIGI	OGTT	Fasting
Osteoglycin pg/ml	2256 (1368;3145)	2273 (1435;3111)	2120 (1417;2823)
p-glucose, mmol/l	5.09 (4.85;5.33)	5.15 (4.73;5.56)	n/a
p-insulin pmol/l	37.2 (21.2;53.3)	31.1 (11.0;51.2)	31.0 (16.6;45.3)

*OGTT* oral glucose tolerance test, *IIGI* intravenous isoglycemic glucose infusion

Likewise, we observed no significant changes neither in percentage s-osteoglycin levels by time (*p* = 0.11) nor over time by study day (*p* = 0.89).

In a simple linear regression analyses, we observed no significant associations at baseline between levels of s-osteoglycin and HOMA-IR, HOMA-β, p-glucose, p-insulin, s-CTX, s-P1NP, p-GIP, p-GLP-1, or p-GLP-2.

We found no significant associations when examining whether changes from time point 0 to time point 60 min in p-GIP, p-GLP-1, p-insulin, and p-glucose were associated with baseline s-osteoglycin levels or changes in s-osteoglycin from time point 0 to time point 60 min.

## Discussion

In the present study, we report that osteoglycin remains stable for three hours during OGTT, IIGI, and fasting in healthy males. The bone turnover marker CTX displays a circadian rhythm with high levels in the morning and has previously been reported to be reduced by 50% after ingestion of a meal [10, 11]. In contrast, we observed that osteoglycin, which is also secreted from the bone, is a stable marker and is independent of fasting or ingestion of glucose. A previous study on eight healthy males reported a decrease in s-osteoglycin following a meal, although only when preceded by rest. This may be a chance finding as the same study reported no changes in s-osteoglycin neither by exercise nor following a meal after exercise [6].

We have previously reported that p-glucose, p-GIP, p-GLP-1, and insulin increased during the OGTT with peaks after 30 to 60 min [8]. In the present study, we observed no association between the increase in the incretin hormones or insulin and baseline s-osteoglycin levels. This is suggestive that osteoglycin is not acutely affected by glycemic responses.

The current data from studies in humans are conflicting on whether a causal relation between s-osteoglycin levels and insulin production or sensitivity exists. Cross-sectional data on individuals with diabetes mellitus have yielded no association between s-osteoglycin levels and markers of glycemia [4, 5]. In the present study, we found no acute changes in s-osteoglycin levels in three different conditions with different levels of circulating insulin. Another study reported that osteoglycin levels are higher in individuals with diabetes mellitus compared to controls; however, this might also reflect differences in BMI and renal function, as these traits have been associated with osteoglycin levels [3, 5]. On the other hand, osteoglycin decreased following weight loss which does not support that high BMI should cause higher osteoglycin levels in type 2 diabetes [2].

Besides actions on glucose metabolism, osteoglycin may interact with bone turnover and quality, as results from a knockout mouse study reported suppression of bone formation by osteoglycin, and another study suggested that osteoglycin regulates collagen fibrillogenesis [2, 12]. We observed no association between CTX or P1NP and osteoglycin levels, and other studies have also reported that osteoglycin is not associated with bone turnover makers [4, 5]. Furthermore, the production of circulating osteoglycin from muscles may be limited in humans as a previous study showed no acute changes in osteoglycin levels following exercise [6].

Thus, current evidence in humans is limited by cross-sectional data which cannot conclude on causality, acute interventions that do not bring about possible chronic effects, and a weight loss study that cannot be directly interpreted as effects of improved glucose metabolism. Therefore, additional research investigating the action of osteoglycin in humans is needed. We propose that future research focus on examinations of the chronic effects of osteoglycin and identification of factors inducing osteoglycin production in humans.

The strengths of the present study are the cross-over design which allow us to investigate acute changes in osteoglycin by different glycemic challenges. Furthermore, the study days mimicked three different endogenous insulin and incretin responses. The study is limited by a small sample size; however, we observed no trend towards changes in s-osteoglycin levels by the different study days. Furthermore, we have investigated osteoglycin in the circulation and not in the muscle or bone were it is produced. The results are obtained in healthy male subjects and may differ in subjects with diabetes or other metabolic disorders. Lastly, the study can only evaluate acute effects on osteoglycin and not chronic effects.

In conclusion, we found that s-osteoglycin levels were stable for three hours during OGTT, IIGI and fasting in healthy males. Based on our findings, circulating osteoglycin levels may be measured independently of fasting or non-fasting conditions. Furthermore, we observed that circulating physiological levels of glucose and insulin did not affect s-osteoglycin levels.
